# Glycotope Sharing between Snail Hemolymph and Larval Schistosomes: Larval Transformation Products Alter Shared Glycan Patterns of Plasma Proteins

**DOI:** 10.1371/journal.pntd.0001569

**Published:** 2012-03-20

**Authors:** Timothy P. Yoshino, Xiao-Jun Wu, Hongdi Liu, Laura A. Gonzalez, André M. Deelder, Cornelis H. Hokke

**Affiliations:** 1 Department of Pathobiological Sciences, University of Wisconsin School of Veterinary Medicine, Madison, Wisconsin, United States of America; 2 Department of Parasitology, Center for Infectious Diseases, Leiden University Medical Center, Leiden, The Netherlands; Biomedical Research Institute, United States of America

## Abstract

Recent evidence supports the involvement of inducible, highly diverse lectin-like recognition molecules in snail hemocyte-mediated responses to larval *Schistosoma mansoni*. Because host lectins likely are involved in initial parasite recognition, we sought to identify specific carbohydrate structures (glycans) shared between larval *S. mansoni* and its host *Biomphalaria glabrata* to address possible mechanisms of immune avoidance through mimicry of elements associated with the host immunoreactivity. A panel of monoclonal antibodies (mABs) to specific *S. mansoni* glycans was used to identify the distribution and abundance of shared glycan epitopes (glycotopes) on plasma glycoproteins from *B. glabrata* strains that differ in their susceptibilities to infection by *S. mansoni*. In addition, a major aim of this study was to determine if larval transformation products (LTPs) could bind to plasma proteins, and thereby alter the glycotopes exposed on plasma proteins in a snail strain-specific fashion. Plasma fractions (<100 kDa/>100 kDa) from susceptible (NMRI) and resistant (BS-90) snail strains were subjected to SDS-PAGE and immunoblot analyses using mAB to LacdiNAc (LDN), fucosylated LDN variants, Lewis X and trimannosyl core glycans. Results confirmed a high degree of glycan sharing, with NMRI plasma exhibiting a greater distribution/abundance of LDN, F-LDN and F-LDN-F than BS-90 plasma (<100 kDa fraction). Pretreatment of blotted proteins with LTPs significantly altered the reactivity of specific mABs to shared glycotopes on blots, mainly through the binding of LTPs to plasma proteins resulting in either glycotope blocking or increased glycotope attachment to plasma. Many LTP-mediated changes in shared glycans were snail-strain specific, especially those in the <100 kDa fraction for NMRI plasma proteins, and for BS-90, mainly those in the >100 kDa fraction. Our data suggest that differential binding of *S. mansoni* LTPs to plasma proteins of susceptible and resistant *B. glabrata* strains may significantly impact early anti-larval immune reactivity, and in turn, compatibility, in this parasite-host system.

## Author Summary

Early larval stages of the human blood fluke *Schistosoma mansoni* face many barriers in their attempt to establish successful infections within their snail host, *Biomphalaria* spp. The snail's internal defense system represents one such barrier, which includes lectin-like recognition receptors and circulating hemocytes capable of encapsulating and killing invading larvae. Since host lectins likely are involved in early immunorecognition events, the recent identification of specific carbohydrate structures (glycans) shared between larval stages of *S. mansoni* and its host *Biomphalaria glabrata* suggests that larvae may be avoiding immune recognition through a molecular mimicry mechanism mediated by lectin-reactive glycans. Results of the present study support previous findings of extensive host-parasite glycan sharing, and demonstrate that molecules released by *S. mansoni* miracidia during *in vitro* development (larval transformation products or LTPs) selectively bind to plasma proteins, altering their reactivity to various glycan-specific monoclonal antibodies. Moreover, some of the changes in recognized glycans following exposure of blotted plasma proteins to LTP were *B. glabrata* strain-specific. We hypothesize that the differential interaction of LTPs with plasma proteins from different *B. glabrata* strains may play an important role in influencing the efficacy of anti-larval immune reactivity within a given host strain.

## Introduction

Glycans are complex carbohydrate (CHO) chains normally covalently bound to polypeptides, lipids or other carrier molecules. Glycoconjugates such as glycoproteins, glycolipids and proteoglycans represent one of the most prominent classes of molecules exhibited by schistosomes. Schistosome glycans are highly diverse structurally and have been implicated in a variety of physiological processes during schistosome infection of its mammalian host, most notably their involvement in modulating protective immune responses and immunopathology (see reviews [1]–[3]). Similarly glycans are also highly expressed in the free-swimming miracidial and intramolluscan developmental stages of *Schistosoma* spp. as shown by earlier exogenous lectin-binding studies [4], [5], and more recent glycotope/glycomic analyses [6]–[9]. However, despite the presence of diverse glycans associated with the larval surface and its secretions/excretions, their functional significance remains unknown. A popular notion that recently has gained traction in the *Biomphalaria glabrata-Schistosoma mansoni* system poses that larval glycans and/or their associated glycoconjugates may be serving as pathogen-associated molecular patterns (PAMPs) that interact with lectin-like pathogen recognition receptors (PRRs), thereby mediating innate immune responses to invading miracidia (see reviews [10]–[13]). This concept has been incorporated into a proposed mechanism, termed compatibility polymorphism [14], in which it is hypothesized that high molecular diversity in relevant PAMP and PRR systems can provide the necessary variation in receptor-ligand interactions to account for differences in infection rates seen in different snail-schistosome strain combinations [15]. Two candidate gene families that fulfill the basic requirements of exhibiting high molecular polymorphism and potential functional diversity are the fibrinogen-related proteins or Freps, lectin-like proteins in plasma of *B. glabrata* snails [16] and a family of polymorphic mucins from *S. mansoni* (*Sm*PoMuc; [17]). Recent studies have reported the selective ability of *Sm*PoMuc to form complexes with Freps from snail plasma [18], as well as the demonstration of a direct linkage between expression of one *B. glabrata* Frep (Frep 3) and resistance to trematode infection [19], thus supporting a functional basis for the compatibility polymorphism hypothesis. The specific ligands mediating Frep-*Sm*PoMuc binding, however, still remain unknown, although the lectin-like properties of Freps and the fact that *Sm*PoMuc are highly glycosylated point to the likelihood that specific glycan structures may be serving to mediate larval recognition, leading to hemocytic encapsulation and parasite killing typical of resistant host phenotypes [20], [21]. Taken further it may then be predicted that the absence of relevant snail Freps and/or differences in expressed glycans (e.g., based on variable *Sm*PoMuc expression) should lead to the opposite schistosome-snail outcome; that is, larval survival due to nonrecognition by the snail's immune system. Assuming snail-schistosome compatibility involves a diversified lectin-based immunorecognition system, the repertoire of larval-expressed glycans (both qualitative and quantitative) potentially has a direct impact on hemocyte reactivity towards a given parasite within the snail host. Following up on Damian's “molecular mimicry” hypothesis [22], [23], that a parasite constitutively expressing host-like molecules could render the host immunologically “blind” to the parasite's presence, it has been suggested that early invading larvae (miracidia, sporocysts) possessing shared antigenic structures with their host snail may evade recognition by the internal defense system [24], [25]. Although earlier studies demonstrated serological reactivity between larval *S. mansoni* and snail host hemolymph [26], [27], Dissous et al. [28] were the first to show that shared CHOs are represented among those immunoreactive epitopes. Recent structural analyses of N-glycans from *B. glabrata* plasma (cell-free hemolymph) provide definitive evidence that glycan structures, specifically terminal fucosylated LacdiNAc variants and core-linked xylose are shared between *S. mansoni* and its snail host [7]. In follow-up studies using highly specific monoclonal antibodies (mABs) to these, and other CHO epitopes (glycotopes), extensive crossreactivity has now been confirmed between larval glycans and those of various *B. glabrata* tissues [7], [9], [29], notably between host hemolymph and proteins released during larval transformation [9]. During the first hours following miracidial entry into the snail host, a complex molecular interplay takes place in which an array of macromolecules are released during miracidium-to-sporocyst transformation [30], [31]. As a consequence, newly developing primary sporocysts are enveloped in a glycan-rich localized environment comprised mainly of glycoproteins, but also may comprise other glycoconjugates. These larval transformation products or LTPs [31], in addition to serving as a passive source of host mimicked” molecules, also may actively bind snail lectins (e.g., Freps; [16]), thereby blocking lectin reactivity against newly developing sporocysts [32]. Given the possible immune modulating effects of LTPs released at a critical time when schistosome miracidia/sporocysts are in the process of establishing infections in the snail host, the present study investigated the effect of LTP exposure on the profile of shared glycotopes associated with plasma from susceptible (NMRI) and resistant (BS-90) strains of *B. glabrata*. Results of these experiments demonstrate that glycoconjugates released during *S. mansoni* larval transformation significantly alter patterns of shared plasma protein glycotopes by either binding and blocking, or by exposing them, thereby providing a possible mechanism by which molecules released by early developing larvae may impact initial immune interactions at the host-parasite interface.

## Materials and Methods

### Ethics statement

All experimental protocols involving mice and rabbits used in the course of this study were reviewed and approved by the Institutional Animal Care and Use Committee (IACUC) at the University of Wisconsin-Madison under Animal Welfare Assurance No. A3368-01.

### Preparation of snail plasma

Hemolymph was obtained from *S. mansoni*-susceptible and -resistant *B. glabrata* snails (NMRI and BS90 strains, respectively) by the headfoot retraction method [33]. Hemolymph from approximately 150 snails (12–15 mm shell diameter) of each strain were pooled and represented a common source for subsequent Western and far-Western blot analyses. During the course of this study a second replicate pool of hemolymph was obtained from which immunoblot analyses were repeated. Upon collection, hemolymph of each strain was dispensed into 1.5 mL microcentrifuge tubes containing cold, sterile Chernin's balanced salt solution (CBSS; [34]) creating a 1∶1 dilution of CBSS:hemolymph. Tubes were centrifuged at 260× g (Eppendorf 5810R, Hauppauge, NY) for 10 min to pellet hemocytes followed by transfer of the cell-free hemolymph (plasma) to a 15-mL Amicon centrifugal ultrafiltration tube (Amicon Ultra-100 k; Millipore Corp, Billerica, MA) with a nominal molecular weight cut-off of 100 kDa. Tubes were centrifuged at 1600× g for 95 min at 4°C (Eppendorf 5810R). This plasma ultrafiltration step was performed in order to separate the snail hemoglobin (which comprises the predominant protein constituent of hemolymph) from a hemoglobin-depleted lower molecular weight plasma fraction. Fractions were designated “>100 kDa-fraction”, which contained the vast majority of hemoglobin as indicated by its intense red color, and “<100 kDa-fraction”, which typically was colorless. Concentration of the <100 kDa-fraction was then carried out using a 3 kDa MW cutoff centrifugal ultrafiltration tube (Amicon Ultra-3 k; Millipore Corp, Billerica, MA). Protein concentrations were determined using a Nanodrop spectrophotometer (ND-1000; Nanodrop Technologies, Wilmington, DE), followed by addition of protease inhibitors (Protease Inhibitor Cocktail SetIII, EDTA-free; Calbiochem, San Diego, CA) to protect proteins from endogenous protease activities.

### Preparation of *S. mansoni* larval transformation products (LTPs)

Miracidia were obtained from eggs recovered from infected mice provided by the Biomedical Research Institute (BRI, Rockville, MD) and housed at the University of Wisconsin Charmany Instructional Facility. Miracidia were isolated under axenic conditions as previously described [35] and placed into *in vitro* culture in CBSS supplemented with penicillin and streptomycin and 1 g/L each of glucose and trehalose (CBSS+) under normoxic conditions at 26°C. Culture supernatants containing larval transformation products (LTPs) were harvested after 24 hr by filtering through a Whatman Puradisc 0.2-µm syringe filter (GE Healthcare LTD, Buckinghamshire, UK) to remove stray sporocysts, epidermal plates, and any cellular debris. Culture supernatants were then concentrated by ultrafiltration (Amicon Ultra-3 k; Millipore Corp, Billerica, MA) and protein concentration determined using a Nanodrop ND-1000. Following addition of protease inhibitors as described above, aliquots were stored at 4°C if used within a week, or at −80°C for longer storage periods. Before use in far-Western blot analyses, concentrated LTP was diluted in snail Tris-buffered saline (sTBS; 20 mM Tris-HCl, 150 mM NaCl, pH7.4).

### Western and far-Western blot analyses

#### Anti-LTP polyclonal and anti-glycan monoclonal antibodies

Production of the polyclonal anti-LTP antiserum is detailed in Wu et al. [31]. Briefly, acetone-precipitated LTP (250 µg in Freund's complete adjuvant) was injected subcutaneously into multiple sites of New Zealand white rabbits, followed by two booster injections using Freund's incomplete adjuvant (Panigen, Blanchardville, WI). Antiserum, harvested from the highest titered rabbit, was applied to a Protein G/Protein A-agarose column (Calbiochem, La Jolla, CA) to isolate the IgG fraction, which was then aliquoted and stored in 0.1% azide at 4°C. IgG isolated from pre-immune serum was used as a negative control.

Monoclonal antibodies (mABs) specifically reactive to a variety of terminal di-, tri- and tetrasaccharide elements of schistosome glycans (listed in Table 1) were produced and characterized as previously described [36]–[38].

**Table 1:** Glycotope specificities of anti-glycan monoclonal antibodies.

MAB Number	Ig Class	Glycotope Structure	Glycotope Name
259-2A1/273-3F2	IgG3/IgM	GalNAc(β1–4)GlcNAc	LacdiNAc (LDN)
114-4E8-A	IgM	GalNAc(β1–4)[Fuc(α1–3)]GlcNAc	LDN-F
258-3E3	IgM	Fuc[(α1–3)]GalNAc(β1–4)GlcNAc	F-LDN
128-1E7-C	IgM	Fuc(α1–3)GalNAc(β1–4)[Fuc(α1–3)]GlcNAc	F-LDN-F
114-5B1-A/290-4A8	IgG1/IgM	GalNAc(β1–4)[Fuc(α1–2)Fuc(α1–3)]GlcNAc	LDN-DF
114-4D12-A	IgG3	Fuc(α1–2)Fuc(α1–3)GalNAc(β1–4) [Fuc(α1–2)Fuc(α1–3)]GlcNAc	DF-LDN-DF
100-4G11-A	IgM	Man(α1–6)[Man α1–3)]Man(β1–4) GlcNAc(β1–4)GlcNAc	Trimannosyl core (TriMan)
128-4F9-A/291-2G3-A	IgM/IgG1	Galβ1–4[Fuc(α1–3)]GlcNAc	Lewis X (LeX)

Schistosome glycan-specific monoclonal antibodies (MABs) were used in Western and far-Western blot analyses to demonstrate the occurrence of shared glycans on plasma proteins from the snail *Biomphalaria glabrata*. MABs were generated from *Schistosoma*-infected mice and their specificities characterized [36]–[38] by screening a panel of synthetic neoglycoproteins bearing schistosome-related glycan determinants.

#### Western/far-Western blot analyses

As a preliminary step to investigating specific glycotope sharing between *B. glabrata* plasma and larval schistosomes, equal concentrations (15 µg in 18 µL/well) of BS-90 and NMRI plasma fractions were separated by SDS-PAGE and subjected to Western blot analysis using the polyclonal anti-LTP antiserum [31]. Anti-LTP Western blots were performed to show that a broad range of LTP epitopes were shared with *B. glabrata* plasma and these epitopes were similarly distributed between plasma proteins of both snail strains. In addition, to compare the protein content and general protein profiles exhibited by BS-90 and NMRI plasma samples, SDS-PAGE separated plasma from both snail strains were stained with Coomassie brilliant blue and silver stains. These initial protein analyses were done to demonstrate that experiments were being performed on NMRI and BS-90 plasma pools that exhibited similar protein banding profiles.

In order to determine the distribution of specific schistosome glycotopes naturally occurring among *B. glabrata* plasma proteins the following protocol was employed: Plasma samples from each fraction (> and <100 kDa fractions) were loaded onto separate 15-well 12.5% SDS-PAGE slab gels with samples from the two snail strains in alternating lanes. This permitted side-by-side qualitative and semi-quantitative comparisons of NMRI and BS-90 plasma reaction profiles for each antibody. After separation and blotting, nitrocellulose membranes were cut into strips, each containing paired NMRI/BS-90 plasma lanes and MW markers, followed by incubation in blocking buffer (5% BSA in sTBS, pH7.4) overnight at 4°C. Blots were then washed 5× in sTBST (sTBS/0.05%Tween 20), incubated in anti-glycan mAB (1∶100 dilution of 5× concentrated mAB culture supernatants) for 2 hr at 22°C, followed by an alkaline phosphatase (AP)-conjugated rabbit anti-mouse Ig antiserum and finally treated with AP color developing reagents (Pierce, Rockford, IL) to visualize immunoreactivities. Due to the absence of LeX antibody reactivity to native plasma proteins from both snail strains, this mAB served as a negative antibody control.

In conjunction with the above direct assessment of shared larval glycotopes on plasma proteins, the effects of LTP exposure on the profiles of shared plasma glycotopes was determined using a far-Western blot approach [39]. Far-Western blot methods are commonly used to test for protein-protein interactions, although interactions between proteins and other molecules may also be assessed. In our far-Western blot assays interactions between plasma proteins and LTPs were assessed. SDS-PAGE-separated and blotted paired NMRI and BS-90 plasma samples were incubated in blocking buffer overnight, washed, and exposed to LTP (50 µg/mL blocking buffer) overnight at 4°C. This was followed by 5 washes in sTBST and 2 hr incubation in anti-glycan mABs at 22°C. Blots were then processed for glycotope reactivity as described above. Western and far-Western blot analyses (using paired plasma samples) for each of the mABs were repeated 2–3 times with similar results. An assumption is made in these assays that upon transfer of plasma protein to nitrocellulose membranes and removal of SDS, polypeptides will reconfigure to their native conformation. Although we realize that reconfiguration may not occur for all proteins, especially those with disulfide-dependent multimeric structures, it is believed that this approach still provides an accurate picture of putative LTP-plasma binding interactions using a comparative experimental design.

## Results

**Figure 1:**
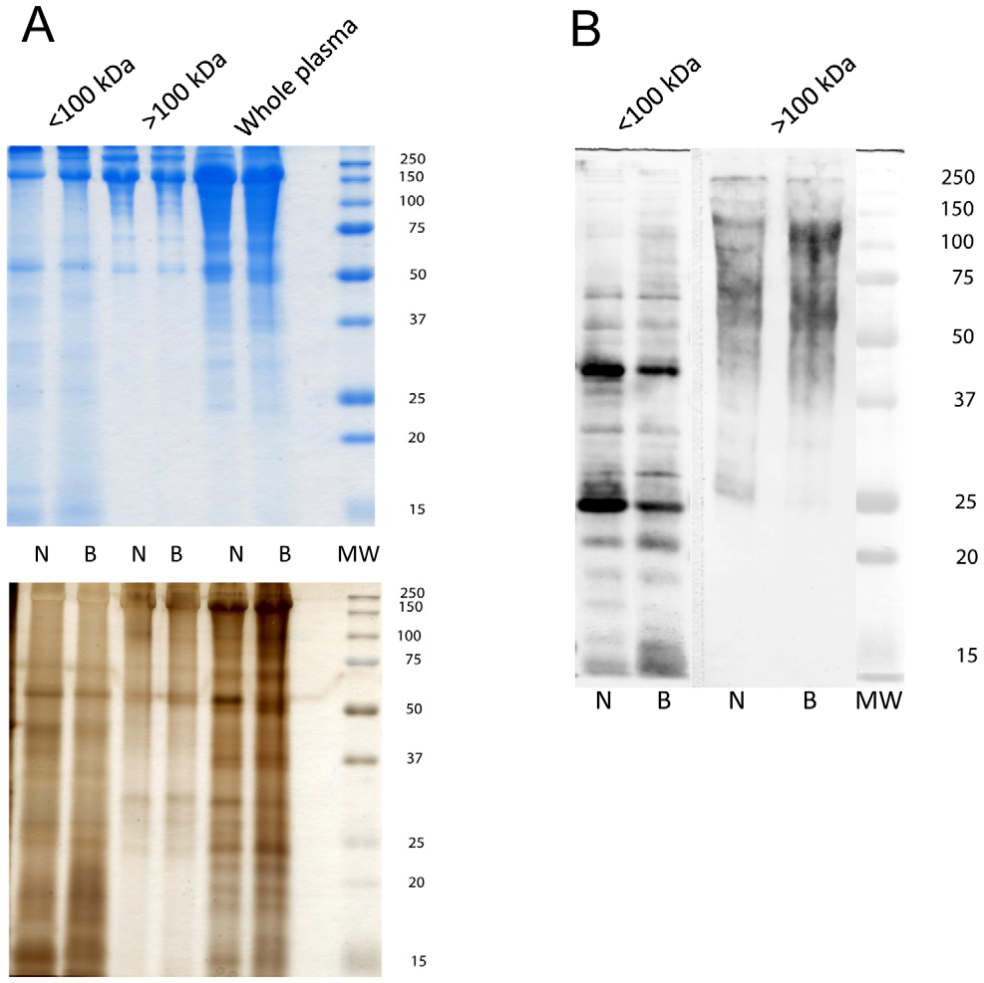
Initial comparison of plasma protein samples from *Biomphalaria glabrata* snail strains. Plasma (cell-free hemolymph) from the NMRI (N) and BS-90 (B) strains of *B. glabrata* was separated into <100 kDa and >100 kDa fractions by molecular ultrafiltration, followed by separation by SDS-PAGE and staining with Coomassie brilliant blue or silver (Figure 1A). Figure 1B is a Western blot of NMRI and BS-90 plasma fractions (</>100 kDa) probed with a polyclonal antiserum to *Schistosoma mansoni* larval transformation products to confirm that epitope sharing in fact occurs between plasma and larval products. Note that plasma from NMRI and BS-90 snails exhibit similar patterns of protein banding (A) and anti-larval immunoreactivity (B) indicating comparable sample loading and the presence of shared epitopes. Molecular weight markers are indicated on the right.

Subjecting *B. glabrata* plasma to centrifugal molecular filtration using 100 kDa MW spin-filters generally was effective in separating intact plasma into fractions enriched for proteins above ∼75 kDa (>100 kDa cut-off) and those of 50 kDa and lower (Figure 1A). Because these ultrafiltration membranes only provide a nominal MW cut-off, clearly there is considerable MW overlap between the high and low MW fractions. The overlap of mid-range MW proteins in both fractions (25–75 kDa) also may have been the result of breakdown of high MW complexes in the >100 kDa fraction during SDS-PAGE processing. Importantly, however, based on protein banding patterns revealed in Coomassie blue- and silver-stained SDS-PAGE gels, only minor differences in protein molecular mass profiles were evident between NMRI and BS-90 snail strains. When separated and blotted snail plasma was probed with a polyclonal antiserum generated against products released during *in vitro* miracidial transformation (LTP), there was extensive immunoreactivity with plasma in both high (>100 kDa) and low (<100 kDa) molecular weight plasma fractions (Figure 1B). Reactivity of our anti-LTP polyclonal antiserum to blotted plasma provided a confirmation that plasma proteins did in fact share crossreactive antigens (epitopes) with products released by *S. mansoni* larvae during early larval development. Because few differences in crossreactive bands were observed between NMRI and BS-90 plasmas using the polyclonal antibody to undefined antigens contained in the LTP, we sought to incorporate highly specific mAB to defined epitopes to identify possible snail strain differences in shared determinants. The similarity in crossreactive plasma profiles also served as an additional sample loading control, allowing for comparisons of mAB reactivities between snail strain samples. These initial analyses of snail plasmas established that (1) NMRI and BS-90 plasma have similar protein content (loading control), (2) that plasma and LTPs did, in fact, share an extensive array of undefined epitopes, and (3) that the use of specific mABs to defined epitopes could provide for a detailed comparison of the shared epitopes exhibited by NMRI and BS-90 snail plasmas in the presence and absence of LTP.

**Figure 2:**
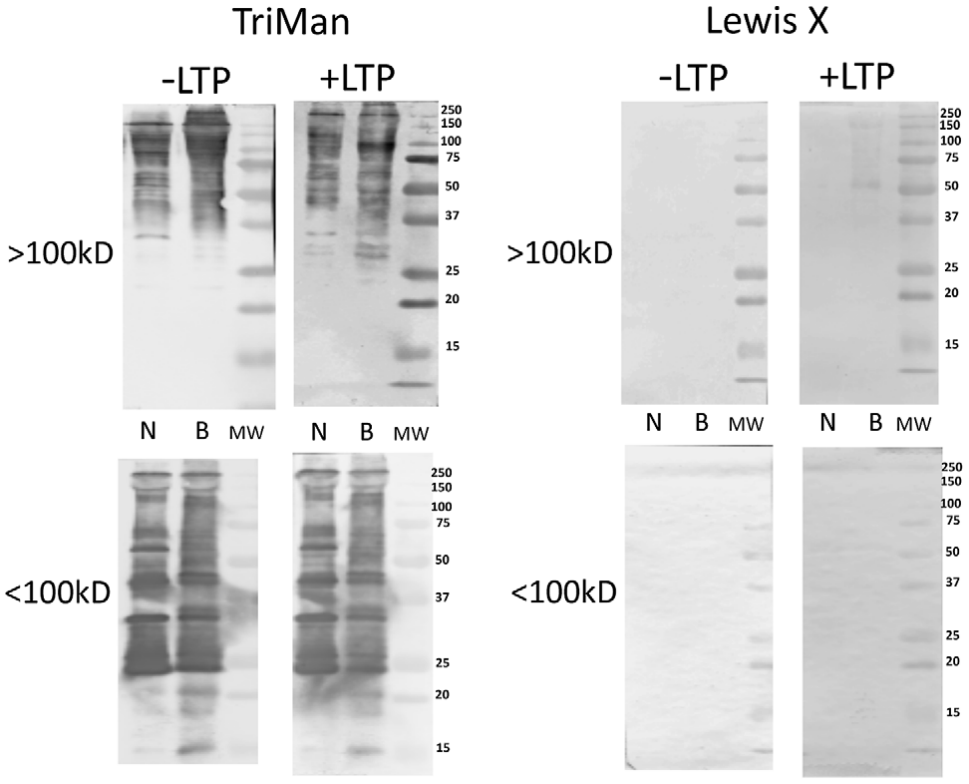
Western/far-Western blot analyses of Lewis X and trimannosyl core glycotopes on plasma proteins. Fractionated *Biomphalaria glabrata* plasma (>100 kDa; <100 kDa) obtained from the NMRI (N) and BS-90 (B) snail strains were separated, blotted to nitrocellulose and pre-incubated in the presence (+LTP) or absence (−LTP) of *S. mansoni* larval transformation products (LTP). LTP-exposed (far-Western) and unexposed (Western) blots were probed with specific anti-glycan mABs against the N-linked trimannosyl core glycan (TriMan) and the Lewis X antigen (see Table 1 for glycan structures). Molecular weight markers are indicated on the right.

In contrast to polyclonal anti-LTP staining, the immunoreactivities of 8 glycan-specific mABs with native (untreated) *B. glabrata* plasma proteins were highly varied as shown by Western blot analyses. Highest reactivity, both in staining intensity and number of proteins, was observed using the trimannosyl N-glycan core (TriMan) mAB, while no staining was seen with anti-Lewis X (LeX) (Figure 2; −LTP). Snail strain differences in staining patterns for TriMan were detected including greater reactivity with NMRI proteins between 50–75 kDa (Figure 2; −LTP/<100 kDa fraction) and in BS-90, proteins between 15–20 kDa (Figure 2; −LTP/<100 kDa fraction). Moreover, pre-incubation of the blotted plasma proteins with *S. mansoni* LTP had little effect on the patterns of shared TriMan glycotopes, with the exception of enhanced staining of BS-90 proteins in the 25–37 kDa range for TriMan (Figure 2; +LTP/>100 kDa fraction). Similarly, interactions of LTP with high MW BS-90 plasma proteins resulted in the appearance of anti-LeX-reactive bands between 50–150 kDa (Figure 2; +LTP/>100 kDa fraction).

**Figure 3:**
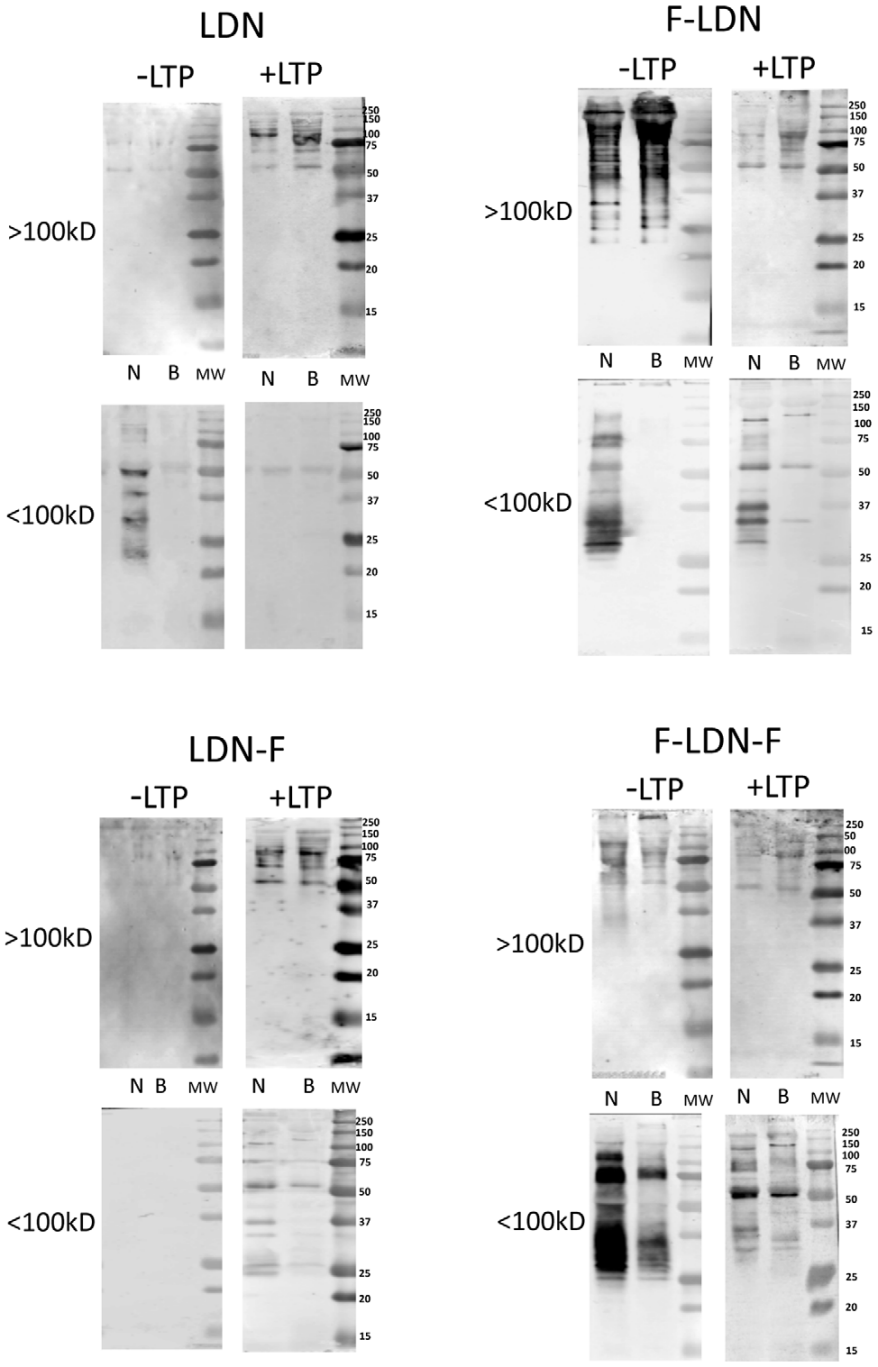
Western/far-Western blot analyses of LDN and (Fucα1,3)-linked LDN glycotopes on plasma proteins. Fractionated *Biomphalaria glabrata* plasma (>100 kDa; <100 kDa) from the NMRI (N) and BS-90 (B) snail strains were probed with anti-glycan mABs recognizing LacdiNAc (LDN) and various (Fucα1,3)-linked forms of LDN (F-LDN, F-LDN-F and LDN-F; see Table 1 for glycan structures and mABs used). Blots containing separated plasma proteins were pre-incubated in the presence (+LTP) or absence (−LTP) of *S. mansoni* larval transformation products (LTP) to determine the effects of LTP exposure on the distribution and intensity of shared glycotopes among proteins of the two *B. glabrata* strains. Molecular weight markers are indicated on the right.

**Figure 4:**
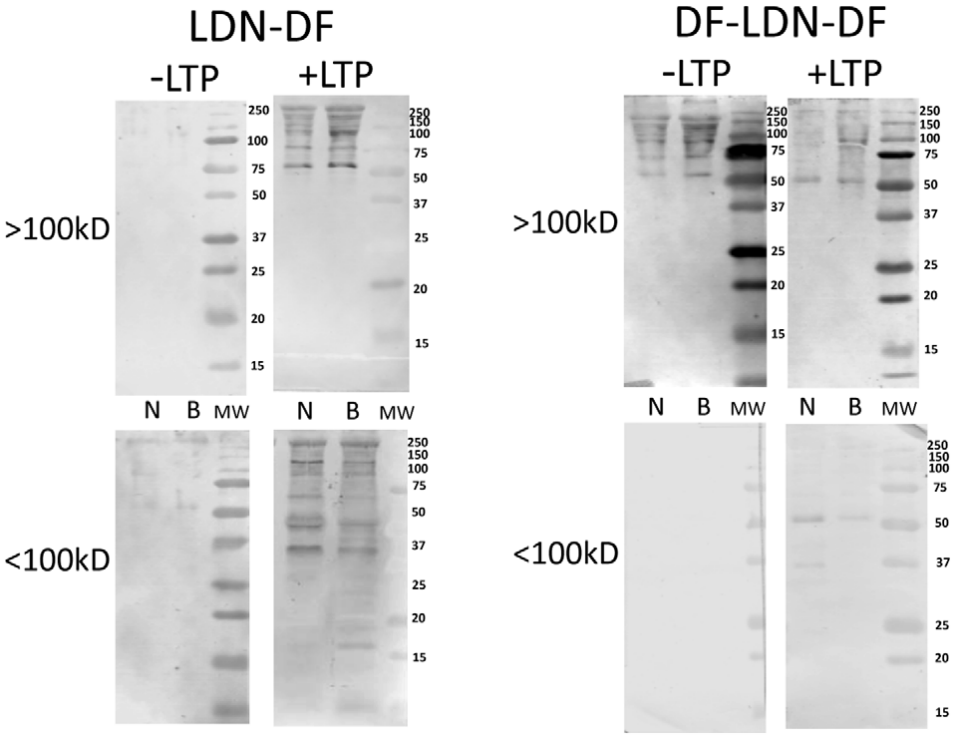
Western/far-Western blot analyses of (Fucα1,2/Fucα1,3)-linked LDN glycotopes on plasma proteins. Fractionated NMRI (N) and BS-90 (B) *Biomphalaria glabrata* plasma (>100 kDa; <100 kDa) were probed with schistosome glycan-specific mABs recognizing LacdiNAc (LDN) containing one or two α1,2-linked fucosyl-dimers (LDN-DF; DF-LDN-DF; see Table 1 for structures and mAB used). Blotted plasma proteins were incubated in the presence (+LTP) or absence (−LTP) of *S. mansoni* larval transformation products (LTP) prior to mAB treatment to determine the effects of LTP exposure on the distribution and intensity of these glycotopes among plasma proteins. Molecular weight markers are indicated on the right.

Similar to TriMan mAB reactivity, consistent qualitative and/or quantitative differences in shared glycotope distribution between native (untreated) NMRI and BS-90 plasma proteins were observed for LDN, F-LDN and F-LDN-F glycotopes, especially in the <100 kDa fraction (Figure 3; −LTP/<100 kDa fraction). Typically, NMRI exhibited higher levels and/or wider distribution of these glycotopes when compared to BS-90 proteins, and notably displayed unique patterns of glycotope reactivity on native proteins indicating that shared glycotopes on NMRI proteins were either associated with unique subsets of glycoproteins or differed quantitatively in their occurrence on individual plasma proteins. Although the occurrence of LDN, F-LDN, LDN-F and F-LDN-F glycotopes varied considerably on plasma proteins contained in the >100 kDa fraction (Figure 3; −LTP/>100 kDa), in the absence of LTP treatment, no consistent snail strain differences were observed for these higher MW proteins. Monoclonal antibodies to LDN-DF and DF-LDN-DF exhibited little reactivity against native plasma proteins, with the exception of higher MW polypeptides in the >100 kDa fraction that stained with anti-DF-LDN-DF (Figure 4; −LTP/>100 kDa). Also, no notable snail strain differences in the distribution of these glycotopes were observed.

Exposure of SDS-PAGE-separated and blotted snail plasma proteins to LTP had a profound effect on the reactivity of plasma proteins to the panel of anti-glycan mABs. Alterations in the exposed glycotope distribution on plasma proteins were manifested in several ways: i) decreased glycotope-specific antibody reactivity in selected plasma proteins as noted with F-LDN, F-LDN-F and DF-LDN-DF (Figures 3 and 4; +LTP/>100 kDa fraction), ii) qualitative changes in antibody reactivity to plasma proteins when compared to non-LTP exposed blots, e.g., F-LDN and F-LDN-F (Figure 3; +LTP/<100 kDa fraction) or iii) appearance of specific glycotopes in previously undetected or existing protein bands. These include the reactivities of anti-LDN-F, LDN-DF and DF-LDN-DF in LTP-exposed plasma proteins in the <100 kDa fraction (Figs. 3 and 4; +LTP/<100 kDa) and anti-LeX, LDN, LDN-F and LDN-DF mAB reactions to the >100 kDa fraction (Figures 2, 3 and 4; +LTP/>100 kDa). Furthermore, it was noted that exposure of blotted NMRI and BS-90 plasma to LTP altered the native (non-treated) glycotope staining profiles for LDN-F, F-LDN, F-LDN-F, and LDN-DF (Figures 3 and 4; +LTP/<100 kDa) and LeX (Figure 2; +LTP/>100 kDa), in a snail strain-specific manner. Examples of plasma proteins that exhibit snail strain-specific changes in glycotope immunoreactivity following LTP-exposure are listed in Table 2.

**Table 2:** Differential effect of LTP-binding on plasma glycotope immunoreactivity.

Glycotope	NMRI *B. glabrata*		BS-90 *B. glabrata*	
	<100 kDa	>100 kDa	<100 kDa	>100 kDa
LeX	-	-	-	55, 80, 150
TriMan	-	-	-	30, 32, 90
F-LDN	37	-	-	130
LDN-F	25, 37, 100	-	-	-
F-LDN-F	-	-	130	130
LDN-DF	45	-	16, 18, 23	-
DF-LDN-DF	37	-	-	-

Listed are the estimated molecular weights (in kDa) of plasma proteins that exhibit snail strain-specific changes in glycotope immunoreactivity following exposure to larval transformation products (LTP). Table headings “< or >100 kDa” refer to the plasma fractions illustrated in Figures 2, 3, 4, while “Glycotope” indicates the glycan epitope recognized by specific mABs (see Table 1 for glycotope structure). Numbers represent approximate MWs of glycotope-bearing plasma proteins that differ between from NMRI and BS-90 *B. glabrata* snail strains.

## Discussion

As alluded to in the introduction, the importance of glycans as potential PAMPs in this model, and probably other trematode-snail systems, has been highlighted in recent studies focusing on the identification of specific glycan structures associated with early developing larval stages of *S. mansoni* [6], [8], [9], [29]. In the present study, mABs generated from *Schistosoma*-infected mice and reactive for terminal elements of schistosome glycans expressed by miracidial and primary sporocyst stages [6], [8], [9], exhibited extensive reactivity with cell-free hemolymph (plasma) glycoproteins of *B. glabrata*. In addition, based on the plasma protein binding patterns for selected glycotope-specific mABs, clear differences were observed between the NMRI and BS-90 snail strains; most notably NMRI plasma possessed a greater number and/or higher staining intensity of glycoproteins displaying the LDN, F-LDN and F-LDN-F glycotopes than plasma proteins from BS-90 snails. These findings are generally consistent with those reported by Lehr et al. [29], although in the present study no consistent snail-strain differences were observed for shared LDN-F, LDN-DF, DF-LDN-DF on native plasma proteins.

Based on the results of these shared glycotope studies, a main goal of the present work was to determine if molecules released during early *S. mansoni* larval development (miracidium-to-sporocyst transformation) are capable of interacting with plasma proteins, and if so, what effect this interaction may have on the patterns of shared plasma glycans in these *B. glabrata* strains. The initial 24 hours of schistosome larval development is believed to be a critical period in determining the success or failure of establishing infections within the snail host [12]. Therefore, unraveling the complex molecular interactions occurring during this time should provide important insights into the underlying mechanisms of host-parasite compatibility in this model system. Upon penetration of a susceptible snail host, miracidia rapidly (within hours) begin transforming to sporocysts by shedding their ciliated epidermal plates during the process of sporocyst tegument formation [40], [41]. As larvae transform they release a complex mixture of molecules (LTPs), including many glycoproteins [30], [31], [42], which are capable of binding snail hemolymph components [43], [44] and modulating hemocyte function [13], [45]–[47], likely through the action of mimicked CHOs [48].

Since innate resistance to incompatible *S. mansoni* strains is mediated by hemocytic encapsulation, the molecular composition of LTPs confronting immune elements in hemolymph would be predicted to have functional consequences resulting from LTP binding to plasma or hemocyte proteins, thereby interfering with larval recognition mechanisms, or by altering hemolymph glycoprotein structures through enzymatic means. In the present study, we have shown that exposure of plasma proteins to LTP dramatically alters the patterns of anti-glycan mAB reactivity for selected glycotopes naturally-occurring (shared) on snail plasma proteins. The most notable effects of LTP exposure on shared glycotope immunoreactivity include reduced or enhanced immunostaining of existing reactive protein bands (e.g., LDN, F-LDN), and changes in existing patterns of plasma glycotope reactivity that results in the appearance or disappearance of immunoreactive bands as noted for anti-F-LDN-F and LDN-F.

At present the mechanism(s) by which LTPs alter plasma protein glycotope reactivity is not precisely known. However, two of the most likely possibilities include (1) the binding of LTP directly to subsets of blotted plasma proteins that results in either enhanced glycotope display if the bound LTP are glycoproteins, or decreased glycotope reactivity due to LTP binding and blocking mAB-reactive plasma glycans, or (2) the presence of specific glycosidases in LTP preparations that are capable of enzymatically altering glycan structures, thereby potentially changing the pattern of specific glycotope reactivity displayed by plasma proteins. A proteomic analysis of *S. mansoni* LTP recently identified an α-N-acetylgalactosaminidase and an endo-α-mannosidase [31], both of which may have potential for altering or removing non-substituted GalNAc (as part of LDN, LDN-F and possibly LDN-DF) and TriMan structures, destroying their mAB-reactivities in the process. However, although specific glycosidase activities may account for LTP-associated decreases in plasma glycotope reactivity, their presence/activities cannot explain why exposure to LTP often results in enhanced mAB reactivity for selected plasma proteins (e.g., LDN, LDN-F) or variable reactivity for a specific mAB among different proteins in the same plasma sample (e.g., F-LDN-F). Moreover, because the endo-α-mannosidase (pH optimum, 7.0) only cleaves α1–2 linkages of extended oligomannose structures [49], it is highly unlikely to be active against the α1–3 and α1–6 linkages comprising the TriMan N-glycan core. The *S. mansoni* α-N-acetylgalactosaminidase also is not predicted to cleave the β1–4 linkage of LDN, thereby rendering it incapable of altering LDN-associated glycotopes. Finally the pH optimum for this enzyme is between 4.3–4.8 [50] and likely would not be active at the pH of the LTP incubation medium (pH 7.4). Therefore, based on the current data, we hypothesize that the direct binding of specific LTPs to selected plasma proteins is responsible for the observed changes in plasma glycotope staining patterns. At this point, however, we do not know to what extent this LTP-plasma binding is due to protein-CHO or protein-protein interactions. Because LTPs appear to bind selectively to subsets of plasma proteins, and in many cases resulting in significant increases or decreases in shared glycotopes associated with a LTP-protein complex, one can imagine a scenario in which miracidia undergoing transformation within the snail host release LTPs that selectively bind to plasma proteins that may be serving as PRRs. Indeed, many of the changes in plasma immunoreactivity seen in LTP-exposed blots occur in proteins between 50–100 kDa (e.g., LDN, LDN-F, F-LDN-F, LDN-DF), which corresponds generally to the molecular size range of the major *B. glabrata* Freps [16]. Current efforts are being focused on identification of the host hemolymph proteins involved in LTP binding interactions.

The findings reported here, and in other recent investigations (reviewed in [12]–[14]) present a number of unanswered questions regarding the functional significance of glycans shared between and/or manipulated within schistosome-snail systems. According to the compatibility polymorphism hypothesis detailed by Mitta et al. [14], the maintenance of high molecular polymorphism (genetic diversity) in both host PRRs (e.g., lectin-like proteins) and their corresponding parasite PAMPs (e.g., glycan-bearing polymorphic mucins) may serve as a possible co-evolutionary mechanism driving the compatibility/incompatibility phenotypes observed in this, and other snail-trematode systems. Although the mechanisms by which snail hemocytes first recognize invading schistosome larvae (including both cellular attraction and adhesion/encapsulation) are still unknown, members of a highly diversified family of fibrinogen-related proteins (Freps) have been implicated as anti-trematode PRRs [19], [51], [52]. Since it has been shown that lectin-like Freps utilize sugars as recognition ligands [19], glycan structures naturally shared between larval *S. mansoni* and host hemolymph or parasite molecules that alter the composition of native glycans displayed by plasma glycoproteins may serve an important role in determining the reactivity of hemocytes towards an individual parasite developing within a given host [12], [13], [24].

With reference to the parasite, genes encoding the polymorphic mucin family (*Sm*PoMuc; [17], [32]) are proposed to provide sufficient genetic diversity to maintain an adequate counterbalance to the highly diversified snail Freps [14]. This is a reasonable notion, especially when one takes into account the added ligand diversity provided by the O- and N-linked glycan structures that can vary greatly, both qualitatively and quantitatively [9], [29]. This glycan diversity was well illustrated in our study showing that, not only are parasite glycans (glycotopes) naturally found on numerous plasma proteins, but snails differing in their susceptibility to the *S. mansoni* strain used in this study, also differed in their repertoires of plasma-associated glycans (e.g., LDN and F-LDN). Moreover, snail-strain differences in the distribution of shared glycans also were noted following LTP treatment of plasma blots (e.g., LeX, F-LDN, LDN-F, LDN-DF).

What is emerging from the present, and other related, investigations is a complex picture of host-parasite molecular interactions that, in accordance with the compatibility polymorphism hypothesis, should provide important clues as to which combinations of host immune factors and corresponding larval factors determine infection success or failure. In support of the above hypothesis, Mone et al. [18] found that fibrinogen-related protein Frep2 bound *Sm*PoMuc in a coimmunoprecipitated complex, which also included, among other adhesion molecules, a Gal-binding lectin. Identifying the repertoire of host immune PRRs (specifically in cell-free plasma and circulating hemocytes) and their specific target ligands (tegumental surface- and LTP-associated glycans) represents the next critical step towards gaining a more comprehensive understanding of the molecular basis for compatibility and incompatibility in this laboratory model of molluscan schistosomiasis. As in mammalian host immune responses to schistosomes [2], [3], it is predicted that glycans, especially those shared with the host, will be shown to play prominent roles as modulators and/or mediators of larval-snail immune interactions.
